# Prevalence of chronic pain in developing countries: systematic review and meta-analysis

**DOI:** 10.1097/PR9.0000000000000779

**Published:** 2019-12-06

**Authors:** Katia Nunes Sá, Larissa Moreira, Abrahão Fontes Baptista, Lin Tchia Yeng, Manoel Jacobsen Teixeira, Ricardo Galhardoni, Daniel Ciampi de Andrade

**Affiliations:** a Escola Bahiana de Medicina e Saúde Pública, Departamento de Pós-Graduação, Pesquisa e Inovação, Salvador, Bahia, Brazil; b Pain Center, LIM62 Neurosurgery, Department of Neurology, University of São Paulo, São Paulo, Brazil; c Universidade Federal do ABC, Centro de Matemática, Cognição e Computação, São Bernardo, São Paulo, Brazil; d Pain Center, Instituto do Câncer do Estado de São Paulo, São Paulo, Brazil; e Instituto de Ortopedia e Traumatologia, Division of Physical Medicine and Rehabilitation, University of São Paulo, São Paulo, Brazil; f School of Medicine, University of City of São Paulo (UNICID), São Paulo, Brazil

**Keywords:** Chronic pain, Prevalence, Review, Meta-analysis, Developing countries

## Abstract

Chronic pain (CP) is prevalent worldwide. Current reports on its prevalence in developing countries are heterogeneous, and to date, there is no quantitative synthesis providing a general estimation of its magnitude in the developing world. The goal of this study was to estimate the pooled prevalence of CP in the general population in developing countries. This was a PROSPERO-registered CRD42019118680 systematic review including population-based cross-sectional studies on CP from countries with ≤0.8 human developing index. We calculated prevalence using both random effects and fixed effects. Heterogeneity was calculated by the Cochran Q test and the I^2^ statistic. Publication bias was evaluated by visual inspection of the Egger funnel plot, as well as by the Begg rank test and the Egger linear test. Sources of heterogeneity were also explored in subgroup analyses. Twelve studies with a total of 29,902 individuals were included in this meta-analysis, of which 7263 individuals were identified with CP. The overall pooled prevalence of CP after correction for publication bias was 18% (95% confidence interval: 10%–29%), the sample presenting significant heterogeneity (I2 = 100%, *P* < 0.001). Subgroup analyses demonstrated that year of publication and the adopted threshold for pain chronicity could partially explain the observed heterogeneity (*P* < 0.05). The proportion of individuals with CP in the general population of developing countries was 18%. However, reports of prevalence have high variability, especially related to year of publication and the threshold level adopted for pain chronicity.

## Abstract 2

Supplemental Digital Content is Available in the Text.

## 1. Introduction

Chronic pain (CP) ranks among the most prevalent medical conditions affecting humans, being among the 10 most prevalent diseases worldwide. Chronic pain is mainly represented by tension-type headache and migraine.^18^ Similarly, when examining the number of years lived with disability (YLD) due to every single disease, low back pain is responsible for the most common cause of age-adjusted YLD in both men and women in most countries. Low back pain and migraine ranked among the top 10 causes of YLD in 195 countries, and neck pain was among the top 5 causes of YLD in high-income and high-middle-income countries.^19,33,38^ Although some pain syndromes are prevalent diffusely worldwide, it has been suggested that regional differences in the prevalence or impact of some CP types could be related to income or related composite measurements (including income per capita, years of schooling, and fertility rates). When looking in detail, the distribution of prevalent types of CP and their respective YLD is not uniform worldwide and does not seem to be monotonically guided by each country's income status. For example, YLD from “other musculoskeletal disorders” were more than twice the rate expected in countries such as Australia, Canada, Chile, and the United States.^27^ Contrarily, Venezuela had less than half the expected rate of YLD from low back pain, and North Korea had more than the double the expected YLD from neck pain.^19^

These data were obtained from both developed and developing countries, and are mainly based on patients who were assisted by medical health care, and had medical outcomes inserted into their national health databases.^39^ This approach is pragmatic and useful, but is clearly affected by access to medical care, regional reporting patterns, and by the mode each disease is handled locally (which may lead to lower or longer YLD). In fact, little is known about the prevalence of CP in the general population in developing countries.^14^ Several studies on the epidemiology of CP in the general population were conducted in outpatient settings, or based on nonrepresentative samples from the population, which could either underestimate or overestimate the actual values of these findings.^27^ A relatively small number of studies have assessed CP prevalence in developing countries, and to date, there are no integrative reviews^31^ assessing the compound prevalence CP in these few available studies. Also, there is currently no systematic assessment of the role of potential influencing variables, such as the definition of CP, and other potential sources of bias such as the year of publication, sample size, or country of origin on the prevalence of CP in these developing areas. Measuring the actual prevalence of CP in developing countries has clear advantages, such as providing supporting information for the guidance of health care policies in these regions, where limited economic resources are the rule. Also, accurate estimates of CP prevalence in economically restricted regions may allow the comparison of regional prevalence findings with data from developed countries, which may support further studies assessing the effects of the potential role of particular variables (higher violence, war, famine, and infectious diseases) on CP prevalence. Finally, having a common denominator of the prevalence of CP in developing areas may serve as a general value against which local prevalence estimates (from a single community, or village, or from 1 particular developing country) could be compared, to classify the local prevalence of CP as lower or higher than expected for areas of similar socioeconomic–demographic backgrounds.^16,29,36^

We have performed the first meta-analysis of CP prevalence of studies from developing countries and have provided analyses on the role of bias and other variables affecting its results.

## 2. Methods

This study was conducted according to the Preferred Reporting Items for Systematic Reviews and Meta-Analyses (PRISMA) (http://www.prisma-statement.org/), and it was registered in the PROSPERO center (https://www.crd.york.ac.uk/prospero/) under protocol number CRD42019118680 on January 9, 2019 (118680).

### 2.1. Study design

We performed a meta-analysis selecting articles reporting cross-sectional CP prevalence of the general population (number of affected persons by the number of exposed) in developing countries.

### 2.2. Search strategy

The search strategy was defined for: (1) PubMed database as a parameter for the others searched databases: (((“Chronic Pain”[Mesh]) OR (Chronic Pain[Title/Abstract] OR Chronic Pains[Title/Abstract] OR Widespread Chronic Pains[Title/Abstract]))) AND (((“Prevalence”[Mesh] OR Prevalences)) OR (“Cross-Sectional Studies”[Mesh] OR Prevalence Studies OR Prevalence Study OR Studies, Prevalence OR Study, Prevalence)) AND ((“0001/01/01”[PDat]: “2017/07/31”[PDat]) AND Humans[Mesh]); (2) Embase database: ((“chronic pain”:ab, ti OR ′widespread chronic pain':ab, ti OR “chronic widespread pain”:ab, ti) AND (“prevalence”:ab, ti OR “prevalences”:ab, ti OR “cross sectional stud*”:ab, ti OR “cross sectional analys*”:ab, ti)) AND “human”/de AND (1982:py OR 1984:py OR 1985:py OR 1986:py OR 1987:py OR 1988:py OR 1989:py OR 1990:py OR 1991:py OR 1992:py OR 1993:py OR 1994:py OR 1995:py OR 1996:py OR 1997:py OR 1998:py OR 1999:py OR 2000:py OR 2001:py OR 2002:py OR 2003:py OR 2004:py OR 2005:py OR 2006:py OR 2007:py OR 2008:py OR 2009:py OR 2010:py OR 2011:py OR 2012:py OR 2013:py OR 2014:py OR 2015:py OR 2016:py OR 2017:py); and (3) Lilacs database: (tw:(“Chronic pain”)) OR (tw:(pain*)) AND (tw:(Prevalence*)) AND (tw:(Developing Countr*).

### 2.3. Eligibility criteria

#### 2.3.1. Types of studies and participants

We included cross-sectional population-based studies enrolling adults (older than 15 years) to study CP (as defined by the respective authors), with a minimum of 100 participants, from countries with ≤0.8 human developing indexes according United Nations Development Program (available at http://hdr.undp.org/en/composite/HDI).

### 2.4. Comparisons

The prevalence of CP was calculated based on the number of individuals with CP and estimates of the size of the general population of each region/country.

### 2.5. Outcomes

The primary outcome was prevalence value and respective confidence interval (CI).

### 2.6. Information sources

**Figure 1.**
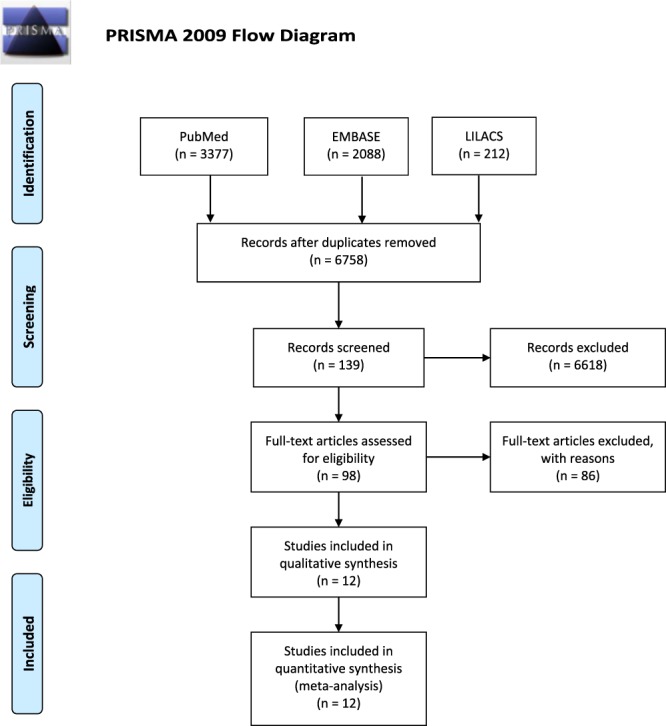
PRISMA flow diagram.

We searched for references to PubMed, Lilacs, Embase, and The Cochrane Library, from inception to November 2018, without limitations idiom (Fig. 1).

### 2.7. Data extraction

We extracted the following information from each of the eligible articles: author's name, publication year, age range, mean age, number of female participants and male participants, location where the study was conducted, population type (general or other), type of interview (face-to-face or telephone interview), sample size, sociodemographic data (when available), number of participants reporting CP and its prevalence with 95% CIs (when not provided it was calculated using the number of individuals with CP based on the percentages), definition CP (3 months, 6 months, or other), average pain duration and intensity (when available), and most frequent pain location and caused (when available). Two studies did not report the number of individuals with CP, only percentages.^11,28^ In these cases, the number of individuals with CP was calculated based on the percentages, and the total number of individuals recruited. Participation (high, moderate, or low) and outcome biases (high, moderate, or low) were based on ranking made by 2 authors (K.N.S. and D.C.d.A.) and are provided as supplementary material (available at http://links.lww.com/PR9/A56). In brief, risk of bias was based on the presence of the following information in the studies: (1) *risk of study participation bias*: Gradings for study participation bias were based on information on the target population, sampling frame/method to assess CP, clear information on the criteria participants needed to fill to be included in the study, and information on study participation/nonparticipation. We assessed information on the reporting of sampling (method used to choose the geographic sampling area, the specific household, and the particular individuals to be interviewed), as well as the number of trials allowed to contact a specific selected household and the reporting of strategies to mitigate nonresponder bias. (2) *Risk of outcome measurement bias:* We looked for information on the use of a clear definition of CP (ideally referenced, with no intrinsic contradictions, and anchored on specific time frames). We assessed whether data collection staff had standardized approach to data collection and followed predefined routines/had standardized files and the use of direct questioning participants instead of having 1 household member reporting pain from other not directly assessed members. Also, we looked for information on the presence of a pilot study, use of double-check assessments to have a reliability measurement of data collection, and presence of estimation of population parameters (estimation of population parameters should emanate from the whole sample and not from subsamples).^30^

### 2.8. Statistical analyses

All statistical analyses were performed using the statistical software R version 3.5.2.^5,37^ Our exploratory analysis started with a visual exploration of all variables to evaluate their frequency, percentage, and near-zero variance for categorical variables, meaning when a categorical variable (eg, country and interview type) had a small percentage of a given category. We also evaluated distribution for numeric variables (such as sample size) and their corresponding missing value patterns. Comparisons for the exploratory analysis were conducted through analysis of variance (*t* tests being a category of analysis of variance) and chi-square tests (the Fisher exact test when any cell presented a frequency below 5). The pooled prevalence of CP was estimated with the R packages “meta” and “metafor.”^6,26,37^ We initially reported a random-effects model, given the expected heterogeneity among studies associated with the diverse settings in which they were conducted. We then compared these results with those from a fixed-effects meta-analysis. We used the inverse variance method to calculate the overall proportion of CP from studies reporting a single proportion, as this is the most widely used pooling method for prevalence meta-analyses.^1^ To reduce issues in the weighting of studies with prevalence close to 0.1, we applied the Freeman–Tukey double arcsine transformation to the individual studies' proportions before calculating the overall proportion.^30^ To calculate CIs for individual study results, we used the exact Clopper–Pearson interval.^6^ To estimate the between-study variance τ2, we used the restricted maximum likelihood estimator as it is considered unbiased and efficient. We evaluated heterogeneity using the Cochran Q test, quantifying it through the I^2^ statistic. Given its known low power to detect heterogeneity, *P* values above 0.10 were deemed as significant for the Cochran Q test. We evaluated publication bias by visual inspection of the Egger funnel plot, as well as by the Begg rank test and the Egger linear test, with a significance threshold of 0.10.^2,9,12^ First, we present the forest and funnel plots for the raw estimates, followed by the results obtained through the “trim-and-fill” method. When asymmetry was identified, we used the “trim-and-fill” method to verify the correction effect on publication bias.^9,12,21,30,41^ We then identified the asymmetry in the funnel plot, followed by the removal of the studies responsible for the asymmetry. The pooled estimate with the remaining studies was calculated, and a new funnel plot was generated by replacing the removed articles and adding their mirror images in the plot. The final pooled results come from an analysis using all true estimates and the simulated mirror images. Finally, we performed subgroup analyses to explore possible sources of heterogeneity based on a wide range of categories: (1) year of publication (2007–2010, 2011–2014, and 2015–2017), (2) geographic region (South America, including Brazil^4,7,17,35,42^; Asia, including China, India, Iran, Nepal, and Philippines^3,11,23,28,43^; and Africa, including Libya and South Africa^13,22^; (3) type of interview (face-to-face or telephone interview),^32^ (4) sample size (lower than 1000, 1001–2000, and greater than 2000), (5) participation bias (low, moderate, and high), (6) outcome bias (low, moderate, and high),^2,12^ and (7) threshold adopted for pain chronicity (pain duration for 3 or 6 months).^18,38^

## 3. Results

### 3.1. Search results

**Figure 2.**
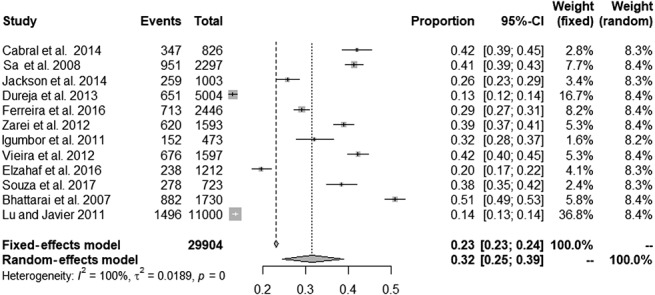
Forest plot presenting the overall prevalence of chronic pain.

After consulting an expert librarian, 2 researchers (K.N.S. and L.M.) independently found 3377 articles in PubMed, 2088 in Embase, and 212 in Lilacs. Of those, 5133 were excluded because of duplication. The application of the screening criteria provided 98 full texts for assessment. On the final analysis based on eligibility criteria, 12 studies with a total of 29,879 individuals were included in this meta-analysis, of which 7,293 individuals had CP (Fig. 2).

### 3.2. Study characteristics

Table 1 displays the overall characteristics of the studies included in the meta-analysis. Selected studies were published between 2007 and 2017. Sample sizes ranged from 473 to 11,000, with a total of 29,904 individuals, of which 7,263 had CP. Geographic locations included South America (n = 5),^4,7,17,35,42^ Asia (n = 5),^3,11,23,28,43^ and Africa (n = 2).^13,22^ All studies targeted the general population, with 7 being conducted by face-to-face interviews and 5 through telephone interviews. Regarding the risk of bias, most studies were classified as presenting high outcome bias (41.67%) or moderate participation bias (58.33%).

**Table 1:**
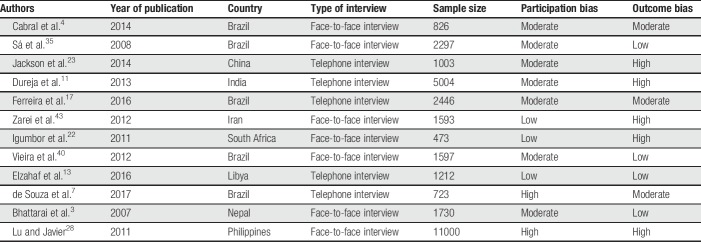
Study characteristics.

### 3.3. Pooled prevalence of chronic pain

**Figure 3.**
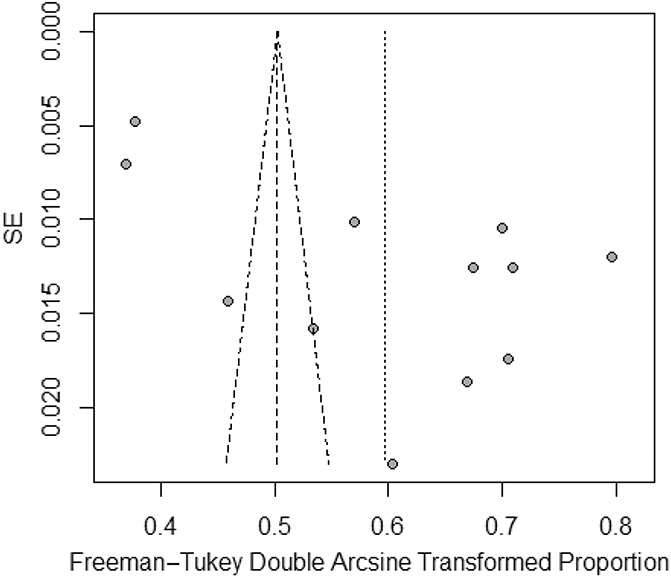
A Egger funnel plot representing the 12 studies included in the meta-analysis.

Figure 3 presents the forest plot with the proportion results for different studies and the overall effect under fixed- and random-effects models, along 95% CIs. The prevalence of CP reported in eligible studies ranged from 13%^11^ to 51%,^3^ being 32% (95% CI: 25%; 39%) using a random-effects model and showing significant heterogeneity (*P* < 0.001, I2 = 100%).

### 3.4. Publication bias

**Figure 4.**
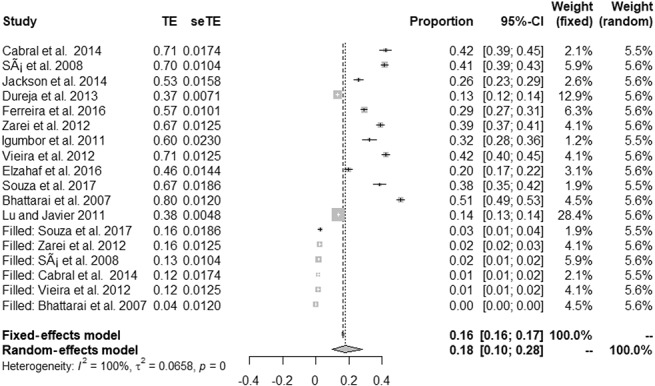
Forest plot presenting the pooled prevalence of chronic pain after adjustment for publication bias.

**Figure 5.**
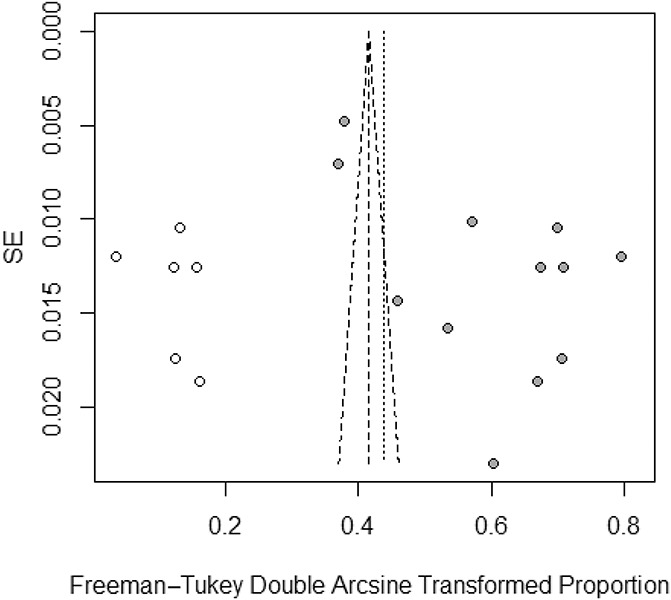
Funnel plot adjusted using the trim-and-fill method with black circles representing comparisons included and white circles representing inputted comparisons using the trim-and-fill method.

Although the results of the Begg rank test (z = 0.274, *P* = 0.784) indicate a low probability of publication bias, results from the Egger linear test (t = 3.490, *P* = 0.005) indicated otherwise. Consistent with the results from the Egger linear test, the Egger funnel plot was asymmetrical (Fig. 4). We therefore used the trim-and-fill method to adjust for publication bias and examined its effect on the pooled estimate. Figure 5 presents the forest plot with the pooled prevalence adjusted for publication bias, which is the prevalence that should be considered for clinical purposes. Figure 5 demonstrates a symmetrical Egger funnel plot after adjusting for missing studies using the trim-and-fill method. Thus, the pooled prevalence of CP, according to a random-effects model, was 18% (95% CI: 10%–28%). Figure 5 demonstrates a symmetrical Egger funnel plot after adjusting for missing studies using the trim-and-fill method.

### 3.5. Subgroup analyses

To explore possible sources of heterogeneity, we further performed subgroup analyzes for the following categories: year of publication, geographic region, type of interview, sample size, participation bias, outcome bias, and CP definition (ie, 3 vs 6 months) (Table 2). Significant between-subgroup differences were observed for year of publication (*P* = 0.016), and for CP definition (*P* = 0.010), which could partially explain the previously observed heterogeneity. The pooled prevalence of CP in studies published from 2007 to 2010 was 46.16 (95% CI: 36.88–55.57), from 2011 to 2014, it was 28.81 (CI: 19.53–39.08), and from 2015 to 2017, it was 28.73 (CI: 18.72–39.91). The pooled prevalence for studies that considered the 3-month definition of CP was 27.42 (CI: 18.64–37.18), and for the 6-month threshold, it was 40.50 (CI: 38.75–42.27). Pooled prevalence for other subgroups is presented in Table 2. The heterogeneity was high (I^2^ > 95%) in most subgroups, being lower among studies with sample size below 1,000 (I^2^ = 84.1%) and for those using the 6-month definition for CP (I^2^ = 48.5%).

**Table 2:**
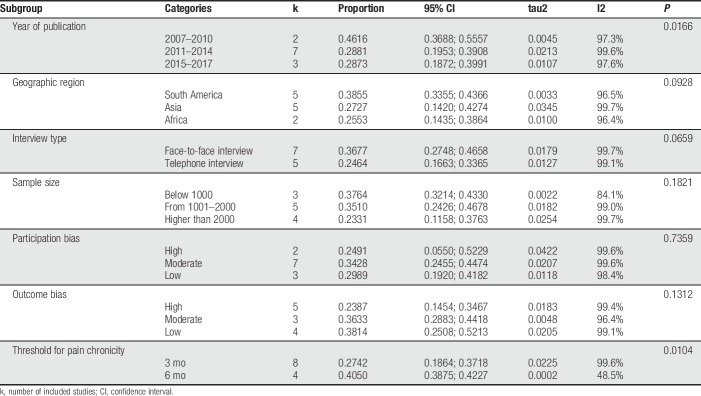
Subgroup analyses by year of publication, geographic region, type of interview, sample size, participation bias, outcome bias, and adopted threshold for pain chronicity.

## 4. Discussion

This is the first meta-analysis specifically studying the prevalence of CP in economically restricted areas of the world. We gathered data from Latin American,^4,7,17,35,42^ Asian,^3,11,23,28,43^ and African^13,22^ countries and found that the prevalence of CP ranged from 13% to 51%. Variable results have also been reported in other studies, ranging from 5.5% to 60.4%, not only for developing but also for developed countries.^10,15,20,34^ This is the reason we chose to adjust our results for publication bias, which has provided us with an actual prevalence estimate of 18%. In fact, other recent studies reporting statistical adjustments for age and sex^14,29,40^ and risk of bias reported values close to the one we found here for developing countries^20,36,39^: In Germany, the prevalence of CP was reported as 18.4%,^20^ while it was 21.5% in Hong Kong,^42^ 24.4% in Norway,^34^ 19% in Denmark, and 19%^15^ and 20.4% in the United States.^25^

In this study, the included studies were published in the last 15 years. We found a great heterogeneity in the definition of CP in developing countries, which is of paramount importance. Although the current definition of CP by the International Association for the Study of Pain (IASP) is that of pain that lasts or recurs for longer than 3 months,^18,38,39^ the actual case definition used by the studies was very heterogeneous. In some reports, intensity of pain was included in the definition^4,7^; in others, the actual criteria used were very complex, considering that pain should be present not only for the last months but also should necessarily be present for the whole day^11^ during the preceding week^7^ or month.^4^ Ferreira^17^ included the wording “suffering” in the definition, which may have comprehension bias that was, to date, not fully explored. Still, in 1 study,^35^ CP was defined as occurring on any day in the previous 6 months. All this variability can affect results from quantitative synthesis such as this one.^1,24,39^ In this line, we found a significant effect of the CP definition on prevalence results. A cutoff limit of 3 or 6 months was determinant to establish the prevalence in this study. The estimated CP prevalence was 30% lower when using the 3-month definition compared with the 6-month one. This is an original finding and gives further support to the need to have unified cutoff duration of CP definition.^38^

Interestingly, another new finding is the presence of a substantial effect of the year of publication on the estimated prevalence. Studies published between 2007 and 2010 reported significantly higher prevalence of CP compared with those published after this period. It is noteworthy that the global years against pain (IASP initiatives) in older persons (2006–2007) and in women (2007–2008) occurred during this period, and this may have stimulated publications of CP prevalence in developing countries with a focus on the elderly and on women. In fact, 2 large studies published or designed on this period had an important emphasis on these topics.^24,39^ Bhattarai et al. reported a higher prevalence of CP in women and in those older than 30 years.^3^ Sá also reported a higher prevalence of pain in women and older individuals.^36^

In this study, we included all studies reporting data in individuals older than 15 years. Although adulthood is frequently defined by a cutoff of 18 years, there is a great variability in age of inclusion in CP studies in both developed and developing countries.^39^ Because in developing countries, individuals older than 16 years are commonly allowed to get married, live alone, and serve the army, we decided to be permissive and set a low bar for age. Indeed, important studies^3,8^ would have been excluded due to the impossibility of extracting data for individuals 18 years and younger. In other included studies, the age cutoff was actually higher than 18 years, being >30 years for Dureja et al. (2013) ^11^ and >20 years for Sá et al.^35^ and Zarei et al.^43^

The subgroup analysis failed to detect significant effects of other potential variables on the final results. Most of the included studies used telephone interviews rather than face-to-face assessments. Despite the general perception that face-to-face assessments are believed to be more accurate,^32^ we found no significant effect of the assessment method in subgroup analyses. The region of the world data came from—South America vs Asia vs Africa—had no significant effects on the score of outcome and participation biases. Interestingly, the sample size assessment suggested that studies with a higher number of participants tended to provide a smaller prevalence of CP, although this has not reached significance. It is noteworthy that studies with a low risk of bias, such as the 1 conducted in Libya,^13^ influenced the final prevalence result to a much higher degree than studies with larger samples sizes but with higher risk of bias.^28^

There are some limitations in this study. First, we found a high heterogeneity of CP definition, which we tried to mitigate with subgroup analysis. However, the actual change in the estimated prevalence of CP if a standardized definition was used remains unknown. In future studies, the broad diffusion of the new IASP/*ICD-11* classification and definition of CP^38^ might help lessen this type of limitation. Also, despite the presence of 12 studies fulfilling the inclusion criteria for participation, most of them were clustered in Brazil (5 studies, 2 from the same city^4,7,17,35,42^), and in the Middle East/Africa,^13,22^ and different parts of Asia.^3,11,23,28,43^ Other Latin American, sub-Saharan African, and Asian countries were either under-represented, or not represented at all. Despite the fact that our subgroup assessment failed to find a “region” influence on results, future studies from these other regions may unravel local differences in the prevalence of CP that might have been missed here.

In conclusion, the adjusted proportion of individuals with CP in the general population of developing countries is 18%, and thus, findings were influenced by the type of definition of CP and the year the study was published, with earlier studies, and those using the 6-month definition of chronicity tending to overestimate its prevalence.

## Disclosures

The authors have no conflicts of interest to declare.

## Appendix A. Supplemental digital content

Supplemental digital content associated with this article can be found online at http://links.lww.com/PR9/A56.

## Supplementary Material

SUPPLEMENTARY MATERIAL
